# Retraction Note: Deficiency of endothelial sirtuin1 in mice stimulates skeletal muscle insulin sensitivity by modifying the secretome

**DOI:** 10.1038/s41467-026-70970-7

**Published:** 2026-03-23

**Authors:** Qiuxia Li, Quanjiang Zhang, Young-Rae Kim, Ravinder Reddy Gaddam, Julia S. Jacobs, Markus M. Bachschmid, Tsneem Younis, Zhiyong Zhu, Leonid Zingman, Barry London, Adam J. Rauckhorst, Eric B. Taylor, Andrew W. Norris, Ajit Vikram, Kaikobad Irani

**Affiliations:** 1 Division of Cardiovascular Medicine, University of Iowa Carver College of Medicine, Iowa City, IA USA; 2 Abboud Cardiovascular Research Center, University of Iowa Carver College of Medicine, Iowa City, IA USA; 3 Department of Internal Medicine, University of Iowa Carver College of Medicine, Iowa City, IA USA; 4 Division of Endocrinology, Diabetes and Hypertension, Department of Medicine, David Geffen School of Medicine and UCLA Health, University of California-Los Angeles, Los Angeles, CA USA; 5 Boston University School of Medicine, Boston, MA USA; 6 Veterans Affairs Medical Center, Iowa City, IA USA; 7 Fraternal Order of Eagles Diabetes Research Center (FOEDRC), University of Iowa Carver College of Medicine, Iowa City, IA USA; 8 Department of Physiology and Biophysics, University of Iowa Carver College of Medicine, Iowa City, IA USA; 9 Pappajohn Biomedical Institute, University of Iowa Carver College of Medicine, Iowa City, IA USA; 10 FOEDRC Metabolomics Core Facility, University of Iowa Carver College of Medicine, Iowa City, IA USA; 11 FOEDRC Metabolic Phenotyping Core Facility, University of Iowa Carver College of Medicine, Iowa City, IA USA; 12 Department of Pediatrics, University of Iowa Carver College of Medicine, Iowa City, IA USA

Retraction to: *Nature Communications* 10.1038/s41467-023-41351-1, published online 11 September 2023

The Editor has retracted this article after concerns were raised regarding the statistical methodology used in the analysis of several key figures (Figs. 1–6, 2s, 4s, and 5s). Specifically, multiple aspects of the statistical analysis were found to be inappropriate or incorrectly applied - these include the use of t-tests instead of ANOVA, the lack of correction for multiple comparisons, sample size too small for valid statistical testing, technical replicates incorrectly averaged or treated as independent biological replicates, and missing or incomplete raw data for several panels. Post-publication review confirmed the concerns. These issues affect the overall conclusions of the article and are too substantial to be rectified with an erratum. The authors are invited to submit a new version of the article, which will then undergo peer review. Markus M. Bachschmid, Leonid Zingman, Barry London, Young-Rae Kim, Andrew W. Norris, Eric B. Taylor, Zhiyong Zhu, Ravinder Reddy Gaddam, Adam J. Rauckhorst, Ajit Vikram, and Kaikobad Irani disagree with this retraction. Qiuxia Li and Quanjiang Zhang did not state explicitly whether they agree with this retraction. Julia S. Jacobs and Tsneem Younis did not respond to correspondence from the Editor about this retraction.

